# Trial of Augustus P. Biegler, for Infanticide

**Published:** 1857-06

**Authors:** 


### ART. II.—*Trial of Augustus P. Biegler, for Infanticide.*

Dr. Biegler has been a homœpathic practitioner of considerable note in Rochester. On a trial before G. W. Clinton, Justice of the Superior Court of Buffalo, commencing on the 13th of May and terminating on the 20th, he was convicted of manslaughter in the second degree, in procuring an abortion on the person of Amelia Murr, resulting in the death of both mother and child.

Mr. James Humphrey, District Attorney, and Mr. Albert Sawin, conducted the prosecution. The defence was in the hands of Hons. Eli Cook and Solomon G. Haven, of Buffalo, and Alfred Ely, of Rochester.

We shall glve the evidence historically, condensing it so far as we can with due reference to the importance of the case. In doing this, we shall pay no regard to the order of the introduction of the witnesses.

Catherine Murr lives in Rochester, is the mother of Amelia Murr. Amelia went to live with Dr. Biegler, as a servant in his family, in September, 1855, being then 17 years old, worked there eight months, after which she came home and staid two weeks. Then Dr. Biegler came, and said that Amelia should go to a Mrs. Moore's, and learn dressmaking. Amelia did so. A month later, that is late in May, 1356, he came and took her trunks away, saying that Amelia was to board at Mrs. Moore's, and cautioning the family not to visit her there.

The Dr. did not take Amelia to Mrs. Moore's, but to a Mrs. Brown's, where he had her boarded as a wealthy patient, suffering with spinal complaint. Nevertheless, Amelia did for some time go regularly to Mrs. Moore's, and work at dressmaking. This labor was entirely discontinued in July. She remained at Mrs. Brown's until Sept. 3, 1856, her family all the time supposing her to be boarding at Mrs. Moore's. During this time

Dr. Biegler visited her every day, with one or two exceptions. On Sundays he usually came twice. He came every evening, remaining two or three hours in Amelia's room, the door being locked. After Amelia had been there two months, Mrs. Brown suspected her to be pregnant from the appearances of her linen.

On the third of September, Biegler took her away, saying that her parents would be in on the cars that day, and Amelia was to go with them and be married. Amelia next appears in Buffalo on the same or a succeeding day. She applied at the house of Mrs. Mercer, No. 7 Scott street, for board; said that her name was Julia Rosendale, that her husband was in California and would be back in the spring to begin a hardware business in Buffalo. At Mrs. Mercer's she remained until her death.

When Amelia left Rochester, her mother felt suspicious, and asked Dr. Biegler where she had gone. Dr. B. assured her that she was married to one Frederick Rosendale, at Erie, a baker; and was well off and happy A day or two subsequently he came to Mrs. Murr with a letter from Amelia dated at Erie, and stating that she was about to leave for Columbus, Ohio. This letter had no post-mark, but Biegler stated that it had been brought to him by a person from Erie. Mrs. Murr recognized the handwriting of her child, but did not believe the letter. She asked Biegler how far it was to Erie, for if it was not too far she would go and see her, and satisfy herself. Biegler told her that it was more than a thousand miles. (It must be recollected that Mrs. Murr was an old woman, and had been only a year in the country.) After this, Biegler came to her and told her that letters from Amelia to her had been lying in the post-office for eight days. Mrs. Murr said it could not be, for her husband inquired every day. The next day such letters were found in the office; they were subsequently proven to have been mailed at the Rochester office, by the Dr. himself. Mrs. Murr never saw her daughter again until she saw her corpse in Buffalo.

Returning now to Buffalo, where Amelia is living with Mrs. Mercer as Julia Rosendale, we find that Mrs. Mercer knew her to be pregnant, but supposed it to be by the supposititious husband in California. During the time between the 3d of Sept. and the 19th of Dec., Biegler visited her every two weeks as Julia's uncle, "Dr. Rosendale." Sometimes she went out with him, sometimes not.

During this time she was in apparently good health, with some exceptions, which are stated by Mrs. Mercer to have been as follows. She had difficulty in breathing, felt as if she would choke sometimes, had headache, and dimness of vision, considerable œdema of the extremities, and had fallen down a flight of steps two or three weeks previously. So far, these are only ordinary phenomena of pregnancy, except that perhaps the difficulty of breathing was more marked than usual, for she required to be propped up high in bed in order to sleep.

Some light on her condition at this time is thrown by a visit she paid to Dr. George Burwell of this city. Dr. Burwell examined her, and seems to have detected albuminuria and suspected that she would have puerperal convulsions at her confinement.

But aside from this it is proved that Julia left Mrs. Mercer's in ordinary good health on the evening of the 19th of December, in company with her "uncle Dr. Rosendale," saying she was going to see some friends stopping at the Railroad Hotel in Exchange street. What was her condition immediately on arriving, is not known. On the history of the few hours passed there rests the interest of the case.

Almon Kingsbury, the clerk of the hotel, swears that Biegler entered his name as H. P. Biglow, and was assigned to room No. 1, used as a public parlor, though it had a bedroom opening out of it. It is not proven that any other persons were in the room except as mentioned in the evidence. Having engaged his room, "he said he might have to stop two or three days. He then said he would go and get his lady. He came back before bed time, came down stairs after a lamp, took two, said he had his wife up stairs, then went to his room.

"About 12 o'clock he came down; said his wife was quite unwell; she was sitting up and had phthisic or asthma; thinks Biegler took up a hot sling for her; he went back to his room, and came down again half an hour after; said his lady was quite unwell; wished witness to go up and see her; witness went up; she was sitting in a rocking chair—was breathing hard, and heaving; Biegler said she was subject to such turns; very little was said; witness went down stairs. In a short time Biegler came down and said that she was sick, he would take her to her friends; witness sent a man for a hack and went to bed. He had no baggage that witness saw; saw Dr. again next morning between 6 and 7, when he handed witness a package or box; described the box. Witness and Biegler sat at breakfast together. He said he took the lady home; that the lady of the house refused to take her; that he said to her, "Is she not an old friend and acquaintance of yours!" that the lady answered that she was not prepared to receive a sick woman, particularly without money; that he then gave her money. After breakfast he took the box and went away.

"When he first came he wrote his name on the large register. The

'room-book' was produced; witness swore that it was the book, and that a name on it was one that he copied from the register. Identified Biegler as the man."

It is requisite that all possible light should be thrown upon Julia's condition at this time. Daniel Brazill, the porter of the house, testifies that he "helped the Dr. get the woman down stairs. She was in the sitting-room, breathing hard. In getting her down, she bore most of her weight on the Dr. Walked with some difficulty about her hips. The Dr. went away with the carriage, came back again, said the woman's husband was in California, and left in his or her aunt's charge, and he would not like to see anything happen to her. He called for something to drink, and treated three or four boys.

Reed Stafford, the hackman who conveyed them back to the house of Mrs. Mercer, swears that he "helped her into the carriage with some one else. Biegler got in with her. She walked with difficulty and was raised into the carriage. Testified to assisting her in the house, No. 7 Scott street. and that she walked with difficulty, was partly carried. Waited some time for the Dr., finally went into the house. He took Biegler back to the hotel."

At Mrs. Mercer's, Julia was placed in a chair. Mrs. M. says; "The Dr came to witness' room and asked if she was asleep. Asked her to get up and do something for this poor woman, for she was dying, she had such a cold. Got up and went into Julia's room, she was sitting on a chair. Dr. said Julia had a very heavy cold, she had asthma; cases of that kind were dangerous and must be seen to immediately. Told her to build a fire, warm some blankets, and get Julia to bed. Witness told Dr. that if she was so sick he must give her some money. He handed witness six dollars —witness thought it not enough. Dr. then gave her sixteen dollars, and took a receipt. He wrote the direction for a doctor in witness' pocket book, "Dr. N. H. Warner, 362 Washington street." He also wrote his address n the pocket book on the other side. It reads, "Mr. Frederick Rosendale. Rochester, in care of Dr. H. P. Biegler.

"Dr. then left, said he would call again next morning. Witness then undressed Julia, she was too sick to help herself much. She sat in one position, did not move or stir at all, had her head down, her hair hanging loose. She appeared in great pain, breathed very quick and heavy, could hardly draw her breath. She could not help herself into bed, and could not lay down when witness put her there. She was in great pain all night, complained of cold. Next morning Dr. Biegler came again, asked how Julia was, and if we had sent for the Dr.

Biegler then went away, and Dr. Warner was called. Dr. Warner testifies to asthmatic breathing. His attention was not directed to her pregnancy or liability to confinement. He prescribed and left. That afternoon (Saturday) while Mrs. Mercer was getting Julia up to fix the bed, she learned that her vulva was very much swollen. Mrs. Mercer went for Dr. Warner, but his partner, Dr. Kenyon, came. She was then removed into the front room, Dr. Kenyon assisting. She could not stand on her feet, but walked with her feet wide apart. Dr. Kenyon testifies that the labia were swollen very much and roughened. That he introduced his finger, she manifesting pain. Found no signs of labor, but a little, *old* clot of blood came away on his finger. Previous to her removal, she attempted to sit in a chair, but sprang up manifesting pain.

These are the essential particulars of the condition of Julia at the time of her return. On Sunday morning it was found that she had raised blood quite freely during the night. She continued to sink during the day, and with the consciousness of death upon her, maintained an entire secrecy as to the cause of her illness, only saying that she had taken cold. She was asked if she wished to send any message, and did request that "her uncle" be informed by telegraph.

A little after nine she wished a chair placed to her feet, a probable indication of expulsive labor pains. The only circumstances attending the delivery of her child were that, only a Mr. Williams being in the room, she said she felt something give way. Williams went out and called in the women. She grasped their hands, raised up looking wildly, and had something like a convulsion. They looked in the bed, found a dead fœtus between her limbs, born apparently in a natural presentation, covered it up, and half an hour later, at about half-past ten on Sunday night, the poor girl died.

Dr. Kenyon came before her death and removed the secundines. He left, having, for some reason unexplained, cautioned the people to care in washing the child. Williams first sees the child rolled up in a towel under the edge of the bed. Dr. K. unrolled the towel, and Williams saw three marks on one breast, two on the other, one on the thigh, and one on the ankle, at the time Dr. K. removed the towel; thinks the two marks on the breast were on the left side; these marks were oblong, about the size of a three cent piece, all of a size except on the thigh and ankles, they were more like a scratch; saw the same spots when the child was washed.

On his cross-examination he called them flesh wounds. Mary Keeler washed the child and saw marks on the breast "something larger than a darning needle." Mrs. Gilbeıt first noticed spots on the child Monday morning; saw the spots on the side, three in a row, half an inch long, they were straight lines; saw spots on the ankles, the skin was roughed up and hanging. On Monday, Dr. S. E. S. H. Nott, the coroner, saw the bodies and noticed marks on the child. On Tuesday he held an inquest, at which Drs. Warner, Garvin, and Jansen, (a medical student,) were present. All of these witnesses subsequently testified to a superficial examination of the child, in which they saw marks thus described by Dr. Warner. There were several marks upon the chest, two seeming to have been made with a round instrument, one on each side; also, one on the abdomen three quarters of an inch in length and transverse the body, seemed to be through the true skin. Another on the left arm seeming to have been made from below upward. There were some other abrasions about the ankles and wrists. Could not say whether the wounds were made during life or after death. There was an appearance of blood on the wounds which led witness to suppose that they were inflicted before birth, or at any rate before the child was cold. At the same time they made a partial examination of the mother. They found what they considered an inflammatory condition of the organs of generation and bowels, and also lacerations in the vulva, with considerable ecchymosis. On Tuesday Dr. Nott issued his warrant, and Biegler was arrested at Rochester. On Wednesdny, the 24th of December, at the instance of the prisoner, another examination was had at the shop of Kraft & Gray, undertakers. Were present at this examination, Prof. Flint, Prof. Hamilton, and Dr. Ellery P. Smith, as examining physicians. Drs. Garvin and Nott were also present by invitation, and the prisoner was there. Only the cavity of the abdomen had been opened at the first examination. On this occasion it was extended to the chest.

Prof. Flint testifies as to the child that he did not examine it. His attention was particularly directed to the lungs, which he found extensively congested and engorged. This congestion was very uniform. A doubt existed as to how much of it was due to a prolonged process of death. The heart was large, measuring five inches in its longest diameter, and four in its greatest transverse diameter. The wall of the left venticle measured more than three quarters of an inch in thickness. Saw no lymph on the bowels. Was not requested to examine the organs of generation.

Prof. Hamilton testified to the same effect, as to the lungs and heart. Examined the child, found various spots upon it when the cuticle had been raised, produced similar spots himself. Did not see any punctures. At the time, he was satisfied that no punctures existed, but could not say that they did not—that is after other witnesses had testified to finding them in a careful examination. As to the mother, he testified to abrasion of the vulva and ecchymosis then. Saw no less on the womb.

Dr. Ellery P. Smith conducted this examination. He testifies to the lungs very much as Dr. Flint and Hamilton do. He said on the stand: I examined the child for the purpose of finding whether it had received any injuries. I found four or five superficial abrasions upon its body and limbs. There were two on the chest. The others were between the knee and ankle. Found nothing passing beneath the true skin. Saw nothing that resembled a puncture. The cuticle was very soft. A very slight pressure of the finger would produce marks similar to those on the body of the child. There were no wounds on the head.

No other witnesses testified to this examination on the final trial. Shortly after it occurred, Coroner Nott granted an examination to the prisoner, which occupied several days. All witnesses hitherto mentioned were sworn at this time. A degree of discrepancy existed, all testifying alike as to the condition of the lungs, while the punctures seen by all the lay witnesses, and Drs. Warner, Garvin, Nott, and Jansen, are not seen at the second inquiry. In view of this, the district attorney instituted a third autopsy, which began at the Medical College on the 30th of January, three weeks after death, and continued during the afternoons of three days. This was conducted by Profs. White and Hunt, and Dr. A. W. Nichols, Demonstrator of Anatomy. During some part of the time, Drs. Nott, Garvin, and Mr. Jansen, were present. Its result is thus stated in Prof. White's testimony. The child was but slightly decomposed. The scarf skin was easily torn by the nail; stated inferences therefrom. The fœtus had not been dissected previously. It did not much exceed six months in fœtal age. The bones of the head were soft, the spaces between them very large, the eyelids adherent, the membrane closing the pupil was still there, the funis attached near the hips, the measurement of the fœtus was 16 inches in length, 9 of which being in head and body, the remainder below; its weight a little less than 3½ lbs., the weight and length greater than usual at this period, according to European authorities; but these signs are not so fixed as the others enumerated. There were a good many marks on the child, on the breast, ankle, &c.; most of them of doubtful character. The mark which attracted most attention was on the right breast, quarter of an inch in depth, apparently made by an instrument an eighth to a sixth of an inch in diameter; it was a punctured wound, made from above downwards, peeling up the scarf skin and penetrating through the true skin. The part containing this wound was carefully dissected out, an incision was made across it, showing that blood had coagulated in the skin and underneath it to the depth of half an inch. In his opinion this showed that the wound had been inflicted during life, or so soon after death that the circulation had not ceased. The nails were membranous, not horny. The lungs of the child were removed and tested. It had never breathed. In relation to the other wounds on the child, they were not conclusive. Described the post-mortem appearances of the mother. The body was but slightly decomposed. The limbs were swollen below the knee, not much, if any, above. * * * He testified to an important wound on the person of the mother, apparently made by an instrument similar to that which had inflicted the wounds upon the child. Some evidences of incipient inflammation were described in the same location. The intestines in the immediate vicinity were injected, discolored, and had some slight deposits of lymph upon them; also slight adhesions of the intestines to each other; these signs indicating inflammation. Supposed there was a connection between the wounds and the inflammation, as cause and effect. This inflammation had not made much progress.

From the appearance and position of the wounds on mother and child, he could account for them by supposing the use of a long metallic instrument for procuring abortion. Witness gave a theory or statement of how this would be likely to occur. Testified that such a wound as that on the child would necessarily result in miscarriage, at a time varying from one to four, five or six days. * * * * * *

On the dissection of the brain found nothing unhealthy. The lungs were detached; in the cavity of the chest was considerable bloody fluid; a number of incisions had been made in the substance of the lungs; some evidences of old pleural adhesions; the lungs were engorged; this condition was very general, but was most conspicuous on the lower portions. Color was a dark red; lung crepitated less than usual between the fingers; a cut in them was smooth, their specific gravity increased; portions cut from the lower parts floated in water; the entire lungs also floated. They weighed two pounds and six ounces. They were not very friable, and on being torn they did not present the granular appearance of inflammation ef the lungs. Testified that he could not say whether this engorgement of the lungs took place before or after death. Such a condition is often found when death has been slow, or the subject has lain something before examination.

The heart weight 14½ ounces, which is above the natural size. The longest diameter of the heart was 5 inches, the greatest transverse diameter 4 inches, walls of left ventricle ⅝ of an inch, walls of right ventricle a little more than ¼ of an inch. Persons may live many years with such a heart; it might embarrass them in rapid motion; any shock would be more serious, asthmatic breathing is a frequent result of it.

The spleen weighed 6 ounces, health; the liver, 3 lbs. 14 ounces, healthy; the pancreas healthy; the kidneys weighed (the right) 4 oz., the left, 4 oz. 1 dram. Both presented some evidences of Brights Disease—perhaps not more than was due to causes already named.

The existence of this disease was confirmed by the swelling of the limbs. Prof. Hunt and Dr. Nichols testified to the same effect. This examination and its result were kept secret. A son of Dr. Biegler applied to Dr. Nichols for information, offering money for it. He was, of course, refused.

On the 26th January, the body was for the second time disintered, and another autopsy held at the instance of the prisoner. Dr. E. P. Smith testifies: I made a second examination on the 26th January. The bodies were in good preservation, little or no decomposition having taken place. It was made at Kraft & Gray's. Drs. Hamilton, Winne, Flint, Burwell, and myself, were present. I performed the autopsy. The child's breast bone was turned up, and its head was not there. I examined the body of the child carefully for punctures. Had heard the testimony of Drs. Garvin and Warner. We examined the whole surface of the child's body with a magnifying glass, every abrasion being examined with particular care. The only wounds we found on the child were where the chest had been cut through to raise the breast bone. Examined below the nipple, on both sides. Did not discover that the skin had been punctured anywhere. The glass used was of three diameters. We examined the uterus and vagina with lenses, and found no puncture of any description. A long time was spent in this examination. We did not discover that any portion of the skin had been removed by the knife; had there been, we should have discovered it. The breast bone had been turned over. Had heard that Drs. Hunt and White had made an examination. Had not heard that they discovered a puncture. Dr. Winne testifies to the same purport with Dr. Smith, as do also Drs. Hamilton and Burwell, except that these gentlemen say that they did not discover the absence of the piece of skin removed by Prof. Hunt, and go no further except to suppose that an incision made across the breast may have been made through the opeuing left by the piece removed, which would, in the elastic skin of a six months fœtus, be ikely to conceal it. Subsequently, Drs. Hunt, White, and Nichols, testify positively to its removal. This is all the evidence in the case, except the opinions of witnesses, which we have in every instance so far as possible omitted. The only allusion we shall make to opinions will be iu our sketch of the arguments of counsel, or in the charge to the jury by Judge Clinton.

Hon. Solomon G. Haven addressed the jury on the part of the defence. After a brief reference to circumstantial testimony, he left the question of motive entirely to the jury. The evidences of dissimulation by Biegler were before them, but he did not consider them as properly a part of the case, which he wished to try on its real issue ; whether the prisoner did feloniously use instruments on the body of Amelia Muir with the intent to procure abortion. The use of drugs for this purpose was ruled out by the fact that such as Bieglər had from time to time prescribed to her were proven to be harmless.

On the single question of the use of an instrument, the proseeution had produced 27 witnesses; eight or ten of them medical men. He wished to carry the theory of the prosecution and that of the defence along together. They show Amelia in a state of good health at Mrs. Mercer's, that she left the house on the evening of Dec. 19th, and we find her next, at about 11 P. M., sick at the Railroad Hotel; that she was helped into a carriage, taken home, and continued ill through Saturday until Sunday night, when she died. The prosecution begin by the presence of many punctures on the body of the child, they come down to a single wound no bigger than the head of a pin. As to the laceration of the vulva, only one examination was made before the death of the mother; that is made by Dr. Kenyon, and there is no evidence that he discovered it then. His hand was the first on the parts, he oiled his hand, showing that the introduction of it was difficult, and who can tell that it did not cause the laceration? He found no laceration, but it should have been there according to the theory of the prosecution, and must have been occasioned by the birth of the child. Every physician testifies that such laceration is not infrequent at the birth of a first child. But if another cause is sought, is not Dr. Kenyon's hand as large as Dr. Biegler's? Again, it is not proven that there was any soreness of the vulva. Mrs. Mercer calls it swelling, and says nothing about soreness. For weeks her limbs had been enormously swollen all the way up to the abdomen, and the swelling is pıoven to be usual in pregnancy.

Mr. Haven went on to advance a theory as to the cause of Amelia's death. She went out with Biegler on a cold, inclement night; it was raining and freezing as it fell; and was it not to be expected that in her feeble health disastrous results would f llow? Dr. Warner finds her laboring under disease of the lungs; she said nothing about any trouble below. Have we not a plain, palpable state of facts to account for this death? Why look further for a cause? She had difficult breathing before. Dr. Winne tells us the effect of puncturing the sac would be to relieve difficult breathing. Dr. Burwell's evidence shows that, with the foresight of a true physician, five or six weeks before her death he judged from her appearance (œdema) that she would have convulsions when she was confined—as she did have—and cautioned her against the exposure that finally caused her death. She was almost blind, she had headache, sleeplessness, could not rest from difficulty of breathing except almost in an upright position; she had swelled limbs, and had fallen down the steps. In this condition she was exposed to inclement weather. Add to this the post-mortem appearances, the engorged lungs, the enlarged heart, the dropsical pericardium, the Brights disease, and the congestion tendencies of pregnancy. This was her condition at 9 P. M., for Biegler came in on the cars at that time. They went down to the Railroad Hotel, without any secresy, and went into room No. 1, a public reception room for travellers. Would the prisoner have chosen such a public place for the commission of a crime?

About 11 P. M. Biegler tells the inmates of the house that she is very ill. The medical witnesses swear to injurious consequences of this exposure. Biegler was a man of skill. He came here with his credentials from the University of Berlin; is it possible that he would have made such a bungling operation? How could he have wounded the breast of the child and not its head? Dr. White is entitled to a patent for showing how, but Dr. Smith, and Winne, aud Havill, and Knickling, all contradict it. Dr. Rochester is brought here to testify that he has touched the breast of a child with his finger. Was it not a shoulder presentation that he thus touched?

The prosecution say that the puncture on the right side presupposes a puncture of the sac. This part of their case is made to hinge entirely on the College examination, made three weeks after death. The child had been examined twice before. The second examination was made at Biegler's request, open and public, in the light of day. If there were any wounds on the child did *he* not know it? But no wounds are found, Dr. Smith asserts positively that none are there, Dr. Hamilton says that he should not have not thought it possible that any were there, had he not heard afterwards that there were. Why is the College examination so secret, conducted under double locks and double keys, and not a word of its results allowed to be whispered? Dr. Hunt, five minutes before the termination of this case, attempts to explain this secresy; he feels the weight of public opinion crushing him down. There is something rotten in this secresy. They cover up their tracks; they throw away the piece of skin said to contain the puncture, and shut it out forever from future examination; and they steal away the remains of the dead. Dr. Nichols testifies that he now has the head of that child. But even in this examination all the marks on the child have disappeared but one. How do they describe it? Dr. Havill tells us—and he is a man of genius—that such a wound should have been an areola; but no such areola is described by these witnesses.

Mr. Haven then commented on the testimony of Dr. White. In describing the wound upon the child, he mentions no external appearances. A barely perceptible mark upon the skin is fixed upon. No ecchymosis is seen there, but the witnesses rely only on internal appeapances; for had there been any external evidences of violence, the second examination would have shown them. This College examination was conductad, not in the light of day and before witnesses, but in secret, in the gloomy, unanswering walls of that room; with double locks and double keys. Professors Flint and Hamilton, teachers in the same school, were carefully excluded and shut out from a knowledge of what had taken place. Drs. White and Hunt, and Nichols, are honorable and scientific gentlemen. I do not intend to impeach them, but there is something so rotten in this secresy that five minutes before the close of this case, Dr. Hunt comes upon the stand here, and asks for a chance to explain it, at a time where we had no opportunity to meet it. He knew that that an explanation was necessary, that the weight of public opinion was crushing him down. Then there is the fourth examination, in which Drs. Winne, Smith, and Burwell, could find no part missing. Smith testifies that it could not have been removed without his knowledge. Where is this piece Dr. Hunt claims to have thrown away? All these witnesses tell you that the edges of the cuts came in perfect coaptation. I do not intend to infer falsehood, but in one of these examinations everything is open, in the other, everything concealed.

The evidence does not hang together. Mrs. Keeler testifies that she rubbed the skin off the breast of the child in washing it, and the evidence excludes all the spots except one on the breast. Dr. White testifies that this wound *may* have been made just after death; but he covers up his tracks and precludes another examination by throwing the piece of skin away.

Mr. Haven then took up the wound on the uterus, dwelt upon its size, and on the fact that by splitting it up they had forever removed all traces of it. Dr. White, again, says that the hœmorrhage and fluids at the Railroad Hotel might have been absorbed in a napkin or napkins. Common sense teaches that this room was public; open to every one, and that the traces of the crime could not have been thus concealed. In examining Julia's bed, Mrs. Mercer found no traces of labor, no hæmorrhage, only a little water as if an infant had wet it; and in the front room a little streak of blood on the sheet which was probably expectorated. Mr. Haven then summed up the various inconsistencies of the case, and urged the possibility that these wounds might be anomalies, vagaries of foetal life.

In respect to the question of motive, why did the prisoner wait till an improbable and inconvenient time and place to commit this crime? It should have been done earlier. Again preparations had been made for confinement, and the abortion implies an improbable abandonment of that intention.

Mr. Haven closed by a brief review of what he had said, and with a characteristic bluntness, closed his speech without any rhetorical flourish.

### MR. SAWIN'S ARGUMENT.

Mr. Sawin complimented the jury on the calm and sober attention they had bestowed on this case. The general theory of the prosecution is that an instrument was applied to Amelia Murr with an intent to procure abortion, and that its necessary effect was to destroy the child and endanger her own life. Two issues are made by the defence, one that no attempt was made, and the other that, if it was made, Amelia's life was not shortened thereby.

Mr. Sawin then took up the case *seriatim.* He traced Amelia to the Railroad Hotel in good health, she comes away unable to walk. What occurred during that time? Dr. Havill's testimony shows that an abortion at the sixth month is difficult, and that the natural result would be a bruising of the soft parts. She had a dropsical condition of the extremities before, but no one pretends that it had caused, or could cause, such soreness of the labia that Amelia could not sit down in a chair without screaming; or that Dr. Kenyon could not examine the parts without giving her much pain. She did not have this swelling when she went out with Biegler, but only six or seven hours afterward it is there, and no natural cause can account for it. Labor was not a cause, for Dr. Kenyon tells you that not a symptom of labor was present when he examined. He says that the os uteri was roughened on the surface, that the parts were dry, and that a little old, dry, clotted blood came away on his finger; not the fresh blood of the usual show. The next evidence as to the condition of the parts is Dr. Warner. He finds the same swelling after death; Jansen swears to inflammation and laceration of the vulva; and Drs. White and Haven testify that this laceration could not in any probability be caused by the delivery of a half formed, flexible six-months fœtus. On the 24th of Dec., Dr. Flint examines, and tells you of inflammation and swelling at the vulva; and the whole testimony on this point places it beyond a doubt that violence was inflicted on the parts on this Friday night.

There would be a natural expectation that evidences of this violence should be found farther up. Drs. Warner, Garvin, and Jausen, swear that the edges of the os were roughened, (abraded,) and that there were appearances of inflammation.

On the 24th, Dr. Flint does not examine the uterus at all, and Drs. Hamilton and Smith make only a casual examination by candle light, in a cold lumber-room. On the third examination a careful search is made with the aid of lenses, and a wound is found, small, but distinct, on the os uteri. (Mr. Sawin here defended the physicians present on the third examination from the charge of secresy made by Mr. Haven. The examination was secret, by order of the District Attorney, and the results were kept concealed lest Biegler should forfeit his bail and run away.)

At the fourth examination made by Drs. Burwell, Winne, Smith, and Hamilton, this wound was not seen for the reason that it had been necessarily obliterated by Drs. White and Hunt in investigating it. The wound on the os uteri is indisputable. That Biegler is skilful in procuring abortion, is no argument against it. The most skilful criminals leave their marks behind them, and he, without regard for the life of the mother, meant to make this thing certain.

Next we come to the fœtus. Wounds existed upon it. Wounds of some kind are sworn to by every witness, lay or medical. Mr. Sawin summed up the evidence on this point at length, and severely censured Dr. Smith for testifying that the square of skin removed removed on the third examination was not missed at the fourth, and that if any such piece had been removed, he should have discovered it.

Why was not the head wounded? Dr. Rochester's evidence proves, experimentally, that this implies no improbability.

Interference by an instrument, then, is proved. Now, who used the instrument? The most skilful criminals are likely to leave a virtual confession behind them. So here, Biegler has confessed this crime. He writes his name on the hotel register as Biglow and wife, and, in writing hyppocritical letters to Dr. Warner as to the death of his victim, he speaks of Mr. Rosendale, her uncle, throughout as his informant, while the fact stands out in flaring colors that he himself is that uncle Rosendale. If he is such a skilful man, this graduate of the University of Berlin, he knew better than to expose his patient to the inclemencies of such a night when in her delicate health. He called in Kingsbury to be sure, but why? He was afraid that his victim might die there alone with him in that room. But he had no tender regard for her. When the rough hack-driver noticed her dying condition, and volunteered to go for a doctor, Biegler refused to have him do so.

Having settled that violence was used, and that by Bi gler, it remains to consider the effect on the life of the mother. No matter how much disease she had about her, if Biegler shortened her life cne moment he was responsible to the law. If she died of cold, taken by exposure, while on her way to the hotel, he was equally responsible for that, for it was a part of the felonious act. Mr. Sawin then closed by an eloquent sketch of the faithful heart with which this poor girl clung to her destroyer, and of the strange power which he seems to have exercised over her throughout.

(These reports of the arguments of Mr. Haven and Mr. Sawin are mere skeleton notes. Each speech occupied between three and four hours. Judge Clinton's charge was equally lengthy, and only a meagre note of it can be made.)

### JUDGE CLINTON'S CHARGE.

On the 21st of December last, Amelia Murr gave birth to a dead child, and herself died about half an hour thereafter. There has been no attempt to show that—if an abortion *was* procured—it was necessary to the preservation of the life of the mother. If any abortion, then, was committed, it was done feloniously, but in order to find the prisoner guilty, you must be satisfied on five points. First, that an instrument was used; second, that the prisoner was the man who used it; third, that the child was quick; fourth, that there was an intention feloniously to cause the death of the child; and fifth, that the death of either mother or child resulted.

At the outset, Judge Clinton cautioned the jury as to the necessity of diverting themselves of all feeling; the more so as this Amelia Murr had a most sad and tragic history. He then took up the five points, but not in the order laid down above.

1. Was the child living? A week before her death Amelia spoke to

Mrs. Mercer of the vigorous motion of the child. In considering this point, you must regard Biegler as a physician, clothed with reasonable knowledge. As a physician, could he have had any motive for procuring the abortion if the child were not quick. Was he innocent, or had he motives for concealing? Again, if you are satisfied that there were punctures on the body of the child, then may you not also be satisfied that the child was alive when at the hotel? Mr. Haven supposes that the punctures, if there were any, were made in washing the child. Does it or does it not follow from the character of these marks that the child was alive a short time before its birth?

2. Assuming that the child was quick, were the instruments used with an intent to destroy it? A person committing any act is presumed to anticipate the usual consequences. If the natural consequence is to cause death, then the presumption is that such was the intent.

3. If the child was quick, and an instrument was used with an intent to destroy it, was Biegler the man who used it? In answering this, you may properly consider these questions. Had any one else opportunity? Is there any reason to believe that any one else did employ instruments, or had any motive to do so? Had any one else a motive to destroy the mother? Was he the father of the child? The testimony of Mrs. Murr as to Amelia's residence of eight months in the house of Biegler in the capacity of a menial; her subsequent employment at Mrs. Moore's; his arrangement with Mrs. Brown and the concealment of her residence from her parents; the fact of his daily visits there; that Amelia's room was locked when he was with her; that he introduced her to Mrs. Brown as a patient, not disclosing to Mrs. B. the residence of her parents; that there is no evidence that she had any spinal disease whatever; her removal to Mrs. Mercer's; her going there under his sanction by a false name; that in communicating with her parents, he concealed her whereabouts; all these facts are evidence as to the question whether he was the father of the child. You may inquire why he should take this deep interest in the girl? If he was the father, does it disclose a motive—I do not mean an adequate motive—to produce abortion on this girl? Had he so entangled himself by his falsehoods that the death of the mother or child would be desirable? Was it desirable to prevent his exposure to the public and his family as a seducer? Did he show a want of proper care as a physician in taking her out on that wild and stormy night? Did he by that act manifest himself an indifference to the life of mother and child? Again, you may ask what honest motive could have prompted him to take her out to that hotel; an act so dangerous. If he had an honest motive, why should he use a false pretence and palm her off as his wife? And now, if he had a motive and an opportunity, had he the means? Does the evidence satisfy you that a single wire carried in his pocket was a sufficient instrument? Was there anything in his letters to indicate that he knew of an abortion on the girl?

4. The fourth question is—Was an instrument employed? It will not do for the prosecution to show that other means, such as his hand, were used. It must be an instrument. With regard to this, the people must rely on circumstances; on the punctures, if any, on the the lips of the uterus and the body of the child. Unless you are satisfied from all the evidence that it was impossible to produce abortion *without* an instrument, I shall not be surprised if this indictment cannot be sustained unless these punctures are proved. The injuries on the vulva may prove violence, but if the punctures are not proven, they go for nothing, And here, I am sorry to say, that the case develops this strange fact, that from some cause or other—I trust not from any action on the part of our county board of supervisors—when at the outset of this case one careful and strict examination was required, four post-mortem examinations have been made.

The first was on Tuesday Dec. 23. It seems to have been directed principally to the question whether an abortion had been procured—and there it stops. Two days after, at the instance of the prisoner, Drs. Hamilton Flint, and Smith, undertook another. That was also imperfect, confessedly so. Their attention seems to be directed by some one to the chest, principally. They re-examined what had been gone over before, more or less, and there stopped. On the 10th, 11th, and 12th of January, another examination was had at the request of the District Attorney, conducted by Drs. White, Hunt, and Nichols. And I wish to say here, gentlemen, that if there were a thorough and scientific examination among the four, this is the one. Hitherto only the eye had been used, here the lens was called in to the aid of the eye. The fourth examination was made at Kraft's, on the 26th of January, by Drs. Winne, Burwell, Smith, and Hamilton. This also, so far as it went, was thorough and scientific; but the inspection with the lens was confined to the fœtus, the womb was not thus examined. I am sorry to say that things have fallen from counsel on both sides to impress you with an unfavorable opinion of the different medical witnesses. If, gentlemen, you find in this evidence any such unfavorable indications, the court does not share them with you. We are unable to discover that any one of these gentlemen has conducted himself in any manner unworthy a gentleman, or that any one of them would have concealed anything calculated to injure either party, much less is there any reason to suppose that any of them have wilfully falsified the facts.

But the question of these injuries on the child is mooted. (Judge Clinton here reviewed the testimony on this point at great length, and with much care. He said that if the evidence established their existence, it was a question whether it would be reasonable for the jury to reject that evidence on the ground of difficulties inflicting them. He read Dr. Smith's evidence in full, remarking that the prosecution claimed something from it, he did not see exactly what. Having reviewed the first, second, and fourth examinations, he took up the third, made by Drs. White, Hunt, and Nichols.) It would seem strange, indeed, to me, gentlemen, if you should discredit their statement that a piece of skin was dissected out and thrown away. There is nothing tending to discredit it, and if they *did cut it out*, then the evidence of the fourth examination as to the non-finding of wounds on the child, goes for nothing.

It is for you to inquire whether you are satisfied on this evidence that a puncture existed on the womb, or on the child before its birth. If on the child can you have the least doubt as to the intent to procure abortion? If on the womb, does it corroborate this intent? The counsel for the defence claim that the wound could not be inflicted on the breast without injuring the head, and that there should have been a flow of blood on puncturing the sac. (Judge Clinton read the evidence on this point, among it that of Prof. Rochester.) With regard to this character of evidence, if the prisoner has proved an impossibility of touching the breast, ask yourselves has he proved that a flow of blood at all or in large quantity necessarily follows the attempt? Have they shown that the fœtus always presents the head? How far are you permitted to oppose improbabilities against positive evidence?

5. The fifth question remains. Assuming that the first four propositions are proved, did the death of the mother or child result from the attempt. All the physicians say it would be dangerous, and also more so in women suffering under chronic disease. Here you must inquire what was the state of her health when she went to the hotel, and afterward. First did she have Bright's disease and enlarged heart. Bright's disease is not necessarily fatal, but brings on dropsy and other diseases, and may lead to abortion. Did the abortion in this case result from disease? If the puncture of the sac inevitably produces aborti n, can the supposition of Bright's disease as a cause, be anything more than a surmise? Did pneumonia ever supervene and cause the death? If so, the prisoner is not guilty. Now, then, if you are satisfied that the attempt at abortion aggravated her diseases and accelerated death, then he is guilty.

In relation to the opinions of physicians, Drs. White, Hunt, and Nichols, were the first to discover the Bright's disease, and several other unhealthy conditions. They testify that the lungs were not inflamed, that only congestion existed. Dr. Smith hardly thought the condition of the lung sufficient to cause death.

I have thus endeavored to suggest questions to your minds as to the five points necessary to be proven. I can do no more but to say to you that the case is not after all such a wonderful one as to require any departure from the ordinary responsibilities of jurymen. Divesting yourselves of all prejudice, you will sift the evidence as I have laid it before you, and bring in a sealed verdict in the morning.

Some objections were made by the counsel for the defence to portions of this charge.

A juryman inquired if they might separate their verdict, so as to bring him in guilty of the death of the child and not of the mother, or vice versa.

Judge Clinton informed them that in either case the verdict would be the same. The death of either mother or child was sufficient for the indictment.

The next morning the jury came in with a verdict of gnilty. Three days thereafter, Biegler was sentenced to confinement at hard labor in the State Prison at Auburn for the term of seven years. This is the extreme penalty of the law for the crime of manslaughter in the second degree.

*Comments by the Reporter.* Our object in this full history is to place the medical evidence on its proper footing before the proper judges, the medical public. Attempts have been made to place the evidence of different distinguished medical gentlemen in contradiction. We leave it far the reader to judge whether any such charge can be properly made. As we view it, on one point only was there any positive discrepancy. Drs. White, Hunt, Nichols, and Nott, all swear to the removal of a piece of skin from the body of the child at the third examination. Dr. Smith testified that at the fourth examination he did not detect the loss of any such piece, and that it could not have been removed without his missing it. We regard this, not as an intentional charge upon the veracity of other witnesses, but as a *lapsus linguæ* under the excitement of his peculiar position. Aside from this one point, we consider the noble charge of Judge Clinton as a sufficient vindication of the faculty of the Buffalo Medical College from the absurd charge that they testified against each other, or manifested par_ tizan feeling on either side. One question will suggest itself to the mind of the reader. Amelia Murr had made her preparations for confinement, and her pregnancy was not concealed. Why did she depart from that intention? And, again, why should Dr. Biegler, a man supposed to be a skilful abortionist, leave such evident traces of clumsy violence behind him?

We do not suppose that Amelia went to the Railroad Hotel with the intention to suffer abortion. But Biegler had the strongest possible motives for the act, and when there, by persuasion and threats, he brought her to his terms, forced her to his purposes, and in the attempt to introduce an instrument upon a woman only partially consenting and struggling against him, he was compelled to inflict the wound on the os and the lacerations of the vulva. The point of the instrument once within the os, he made sure work and inflicted the punctures on the child. Such is our theory.

There is something strangely pathetic in the constancy of this poor girl to her destroyer. She was beautiful, her form the most magnificent model we ever saw. Her mind was that of a true woman, faithful through much suffering; and the unvarying secresy which she had maintained through all their intercourse found its fitting termination in the closed lips, which, in the agony of death, gave no history of her wrongs, and sent only a thoughtful message of love to the accursed villlain who had robbed her of good name, of parents, home kindred, of her unborn child, and her own existence.

